# Evaluating the efficacy of post-surgery adjuvant therapies used for ductal carcinoma *in situ* patients: a network meta-analysis

**DOI:** 10.18632/oncotarget.17366

**Published:** 2017-04-21

**Authors:** Li Wang, Yaoxiong Xia, Dequan Liu, Yueqin Zeng, Li Chang, Lan Li, Yu Hou, Lv Ge, Wenhui Li, Zhijie Liu

**Affiliations:** ^1^ Department of Radiation Oncology, The Third Affiliated Hospital of Kunming Medical University, Tumor Hospital of Yunnan Province, Kunming, Yunnan, China; ^2^ Department of Breast surgery, the Third Affiliated Hospital of Kunming Medical University, Tumor Hospital of Yunnan Province, Kunming, Yunnan, China; ^3^ Department of Breast Surgery, The Third Affiliated Hospital of Kunming Medical University, Tumor Hospital of Yunnan Province, Kunming, Yunnan, China; ^4^ The Third Affiliated Hospital of Kunming Medical University, Tumor Hospital of Yunnan Province, Kunming, Yunnan, China

**Keywords:** ductal carcinoma in situ, radiotherapy, tamoxifen, anastrozole, network-meta-analysis

## Abstract

**Objective:**

Post-surgery adjuvant therapies are very important for patients suffering from ductal carcinoma in situ (DCIS). In this study we conducted a network meta-analysis (NMA) to evaluate the efficacy of different post-surgery adjuvant therapies including tamoxifen, anastrozole and radiation therapy (RT) and their combinations (RT+ tamoxifen and RT+ anastrozole).

**Methods:**

We searched several databases, including Embase, MEDLINE / PUBMED, Cochrane Library, and Science Citation Index, for relevant studies. We then extracted the data from eligible studies in order to perform our NMA. We measured the comparative efficacy of each treatment option based on the calculated odds ratios (ORs) and the corresponding 95% credibility interval (95%CrI) for each treatment option. We calculated the surfaces under the cumulative ranking curves (SUCRA) in order to rank the therapies according to their different outcomes.

**Results:**

In this study, local recurrence (LC) was chosen as the primary outcome. Metastasis, contralateral-breast cancer (CBC), ipsilateral-breast cancer (IBC) and death were secondary outcomes. Patients treated with RT and RT + tamoxifen exhibited a lower risk of LC compared with control group (OR=0.54, 95%CrI: 0.40-0.73; OR=0.41, 95%CrI: 0.19-0.90). Patients treated by RT and RT + tamoxifen also exhibited a significantly lower risk of IBC compared with control group (OR=0.55, 95%CrI: 0.37-0.82; OR=0.42, 95%CrI: 0.18-0.99). Results from the SUCRA indicated that RT + anastrozole and RT + tamoxifen were potentially the best adjuvant treatments for patients with DCIS.

**Conclusions:**

In conclusion, the RT + anastrozole and RT + tamoxifen are recommended for their performance and effectiveness.

## INTRODUCTION

Ductal carcinoma *in situ* (DCIS) of the breast is the result of the clonal proliferation of malignant-appearing cells contained in the mammary duct lumens. It is a precursor to invasive breast carcinoma [1]. It is estimated that 45-78% of invasive breast carcinomas are associated with DCIS [2]. Although DCIS itself has a relatively low risk of metastasis [3], about 25% to 50% of DCIS patients eventually develop invasive cancers [4]. In cases of invasive relapse, the patient survival rate is about 15% [5]. The discovery of similar chromosomal changes in DCIS and invasive cancers patients suggests a potential relationship between DCIS and invasive cancers [6, 7].

Although DCIS is usually not detectable in its early stages, mammographic screening techniques can be used to detect it before clinical symptoms are generally observable [3, 8]. The number of diagnosed DCIS cases in the US has increased notably after mammographic screening techniques were introduced [9].

Traditional mastectomy is frequently used to prevent local recurrence (LC) in patients with DCIS, however it may cause severe side effects [3, 10, 11]. As a result, the conventional mastectomy has been replaced by breast-conserving surgery (BCS) with or without the use of adjuvant endocrine or radiation therapy (RT) [12]. Randomized controlled trials (RCTs) in previous studies have indicated a remarkable decrease in the risk of LC among DCIS patients who were managed by post-lumpectomy RT [13, 14]. Another (National Surgical Adjutant Breast/Bowel Project) NSABP B-17 trial suggested that the LC rate during an eight-year period decreased to 12% in DCIS patients with postoperative RT [13]. Some consistent results were reported The European Cooperative Study Group Trial (EORTC), reported consistent results in their study of 1,010 females who were diagnosed with DCIS [14]. These trials demonstrated that RT could reduce the risk of LC in DCIS patients.

On the other hand, tamoxifen is often used as an adjuvant endocrine therapy for invasive breast carcinoma and it is able to reduce the risk of LC and death [4]. Moreover, tamoxifen has gradually gained use as an adjuvant therapy for DCIS patients in order to prevent ipsilateral and contralateral failure. In the NSABP B-24 trial, females with tamoxifen therapy following BCS and post-lumpectomy irradiation had significantly fewer breast cancer events than those with placebo during an 83-month follow-up period [15]. The effectiveness of tamoxifen in patients treated by BCS and postoperative RT has been verified and hence tamoxifen is often recommended to DCIS patients in order to prevent invasion [4].

Anastrozole is a non-steroidal aromatase-inhibiting medication. It is also an approved treatment option for breast cancer patients and it has been suggested that it is even more effective than tamoxifen with respect to disease progression and overall response rates [16]. Although anastrozole and tamoxifen have similar side effects and toxicity, patients treated by anastrozole exhibited fewer thromboembolic events compared to those treated by tamoxifen [15]. The ATAC6 trial, which was conducted in females with early-stage invasive breast carcinoma, revealed similar results [17]. In addition, patients treated with anastrozole were associated with fewer cases of deep-vein thrombosis, hot flush, endometrial cancer and stroke compared with those treated by tamoxifen [15].

However, there is no NMA comparing different adjuvant therapies for patients with DCIS. This NMA was conducted to compare the efficacy of various postoperative adjuvant therapies for patients suffering from DCIS in order to evaluate the performance of different post-surgery treatments and provide grounded information for clinical practice. The therapies considered include: tamoxifen, anastrozole, RT, RT + tamoxifen and RT + anastrozole. The control group, set up for the purpose of comparison, consisted of patients treated with placebo. This study used endpoints, including LC, metastasis (M), contralateral breast cancer (CBC), ipsilateral-breast cancer (IBC) and death (D) to assess the efficacy of the above treatments.

## RESULTS

### Literature selection

**Figure 1:**
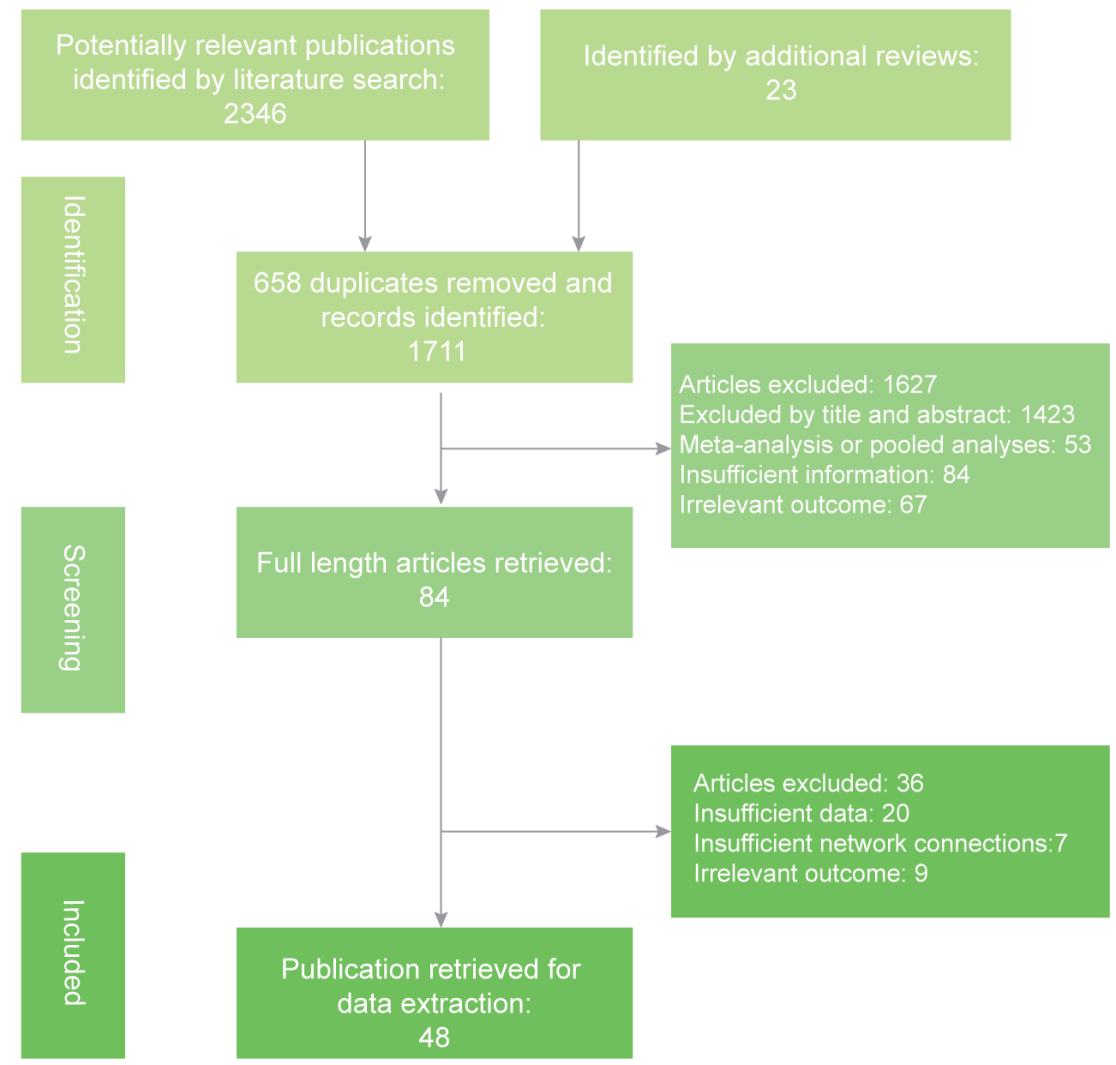
Flow chart of literature search, screening and inclusion

**Figure 2:**
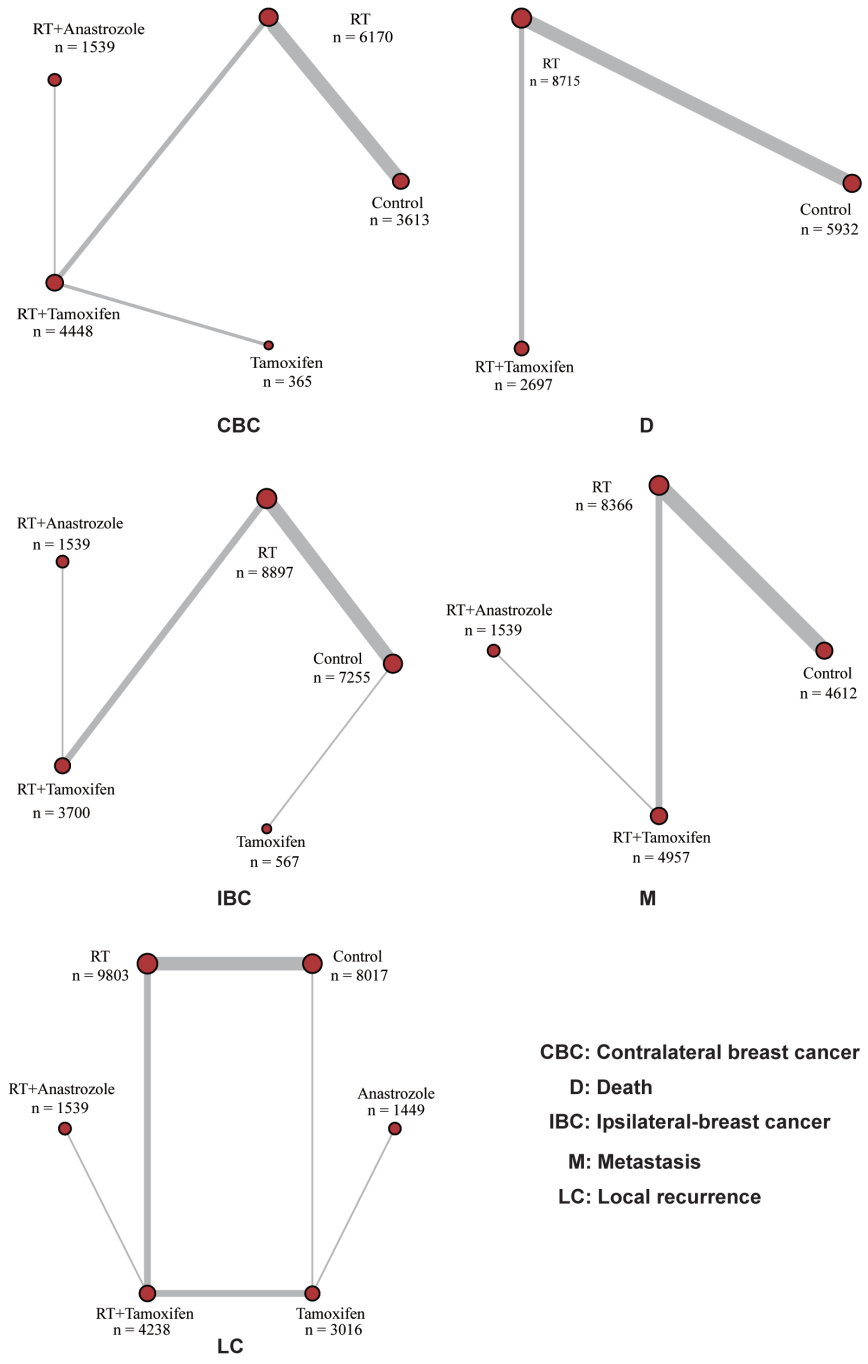
Network plot for each outcome: each node corresponds to an adjuvant therapy and direct comparisons are connected by solid lines

As shown in Figure 1, study selection was carried out in three steps: study identification, screening and inclusion. We identified a total of 2,369 studies, 2,346 of which were identified using the literature search strategy outlines above, and 23 of which were identified by additional reviews. However, only 84 full-length articles were retrieved and the remaining articles were rejected due to duplication, insufficient information or irrelevant outcomes. Another 36 articles were excluded because of insufficient data, insufficient network connections or irrelevant outcomes. Therefore, 48 eligible publications were eventually subject to data extraction [13, 15, 18-63]. The detailed characteristics of eligible studies are displayed in Table 1. Of the 48 eligible studies, 29 were observational studies and the other 19 were RCTs. A total of 41,922 patients were included in our NMA and the corresponding following-up duration of each study ranged from 3 to 20 years. Up to 40 studies covered the comparison of the RT group and control group. The network plot for each outcome is illustrated in Figure 2, in which each node corresponds to a post-surgery adjuvant therapy and each solid line corresponds to a direct comparison between two therapies. The thickness of solid lines is proportional to the number of direct comparisons between two therapies whereas the size of nodes is proportional to the sample size involved in each therapy.

**Table 1:** Baseline characteristics of included studies

Study	Country	Design	Subgroup by	Follow-up (months)	Surgery	Group 1		Group 2		Outcomes				
						Size	Therapy	Size	Therapy	LC	M	CBC	IBC	D
Baird, 1990	Multicenter	nRCT	-	30	Mastectomy	30	Control	8	RT	√				
Ben-David, 2007	USA	RCT	-	48	BCS	48	RT+Tamoxifen	150	RT	√			√	
Bijker, 2006	Multicountry	RCT	-	503	LE	503	RT	507	Control	√	√	√		√
Bijker, 2001	European	nRCT	-	134	LE	134	Control	159	RT		√			√
Boyages, 1999	Australia	nRCT	-	289	BCS	289	Control	357	RT	√				
Cataliotti, 1992	Italy	nRCT	margin status	123	BCS	123	RT	167	Control	√				
Chan, 2001	Multicenter	nRCT	margin status	129	BCS	129	Control	18	RT	√				
				27	BCS	27	Tamoxifen	9	RT+Tamoxifen	√				
Chuwa, 2008	Multicenter	nRCT	margin status	67	BCS	67	Tamoxifen	103	RT+Tamoxifen	√		√		
Cutuli, 2002	France	nRCT	age	515	BCS	515	RT	190	Control	√	√			
Cutuli, 2001	Multicenter	nRCT	age	136	BCS	136	Control	435	RT	√				
Cuzick, 2011	Multicenter	RCT	grade, age	567	WLE	567	Tamoxifen	316	RT+Tamoxifen	√				
				544	WLE	544	Control	267	RT	√				
Di Saverio, 2008	USA	nRCT	age, size of lesion, VNPI	186	BCS	186	Control	73	RT	√				√
Donker, 2013	European	RCT	-	503	LE	503	Control	507	RT	√	√			√
Emdin, 2006	Sweden	RCT	-	526	BSR	526	RT	520	Control	√	√	√	√	√
Fentiman, 1998	Multicenter	nRCT	-	405	WLE	405	Control	413	RT				√	
Fisher, 2007	Multicountry	nRCT	-	734	LE	734	RT	722	RT+Tamoxifen	√	√	√		
Fisher, 2001	-	RCT	-	403	Lumpectomy	403	Control	410	RT		√	√	√	√
				899	Lumpectomy	899	RT	899	RT+Tamoxifen		√	√	√	√
Fisher, 2001	NSAPB	RCT	-	522	Lumpectomy	522	Control	517	RT					√
Fisher, 1999	Multicenter	RCT	margin status	899	Lumpectomy	899	RT	899	RT+Tamoxifen		√	√	√	√
Fisher, 1999	USA	RCT	-	303	Lumpectomy	303	Control	320	RT				√	
Fisher, 1995	Multicenter	RCT	margin status	274	Lumpectomy	274	Control	299	RT				√	
Fisher, 1993	UK	RCT	-	391	Lumpectomy	391	Control	399	RT		√	√	√	√
Forbes, 2016	Multicenter	RCT	invasive breast cancer	1489	LE	1489	Tamoxifen	1449	Anastrozole	√				√
Habel, 1998	Multicenter	nRCT	-	248	BCS	248	Control	293	RT	√				
Holmberg, 2008	Sweden	RCT	age, size of lesion	526	BCS	526	RT	520	Control		√	√	√	√
Houghton, 2003	Multicenter	RCT	age	544	LE	544	Control	567	Tamoxifen	√			√	
				267	LE	267	RT	316	RT+Tamoxifen	√			√	
Jha, 2001	UK	nRCT	-	94	WLE	94	RT	30	Control	√				
Julien, 2000	Multicountry	RCT	-	503	LE	503	Control	507	RT	√	√	√		√
Kestin, 2000	USA	nRCT	-	36	Lumpectomy	36	RT	142	Control		√	√	√	
Kuske, 1993	USA	nRCT	-	70	Lumpectomy	70	RT	7	Control	√				
Lagios, 1989	USA	nRCT	-	230	Lumpectomy	230	RT	101	Control	√				
Lara, 2003	USA	nRCT	-	86	Mastectomy	86	RT	16	Control			√		
Margolese, 2016	USA/Canada	RCT	-	1538	Lumpectomy	1538	RT+Tamoxifen	1539	RT+Anastrozole	√	√	√	√	
McCormick, 2015	Multicenter	RCT	-	298	BCS	298	Tamoxifen	287	RT+Tamoxifen	√		√		
Meijnen, 2008	-	nRCT	-	91	WLE	91	Control	119	RT	√	√	√		√
Miller, 2001	Multicenter	nRCT	-	88	Lumpectomy	88	Control	18	RT	√			√	
Omlin, 2006	Multicountry	nRCT	-	166	BCS	166	RT	57	Control	√				
Rakovitch, 2007	USA	nRCT	age	310	BCS	310	Control	305	RT	√				
Ringberg, 2000	Multicenter	nRCT	grade	66	BCS	66	RT	121	Control	√				
Rudlof, 2010	USA	nRCT	age, number of excision	935	BCS	935	Control	906	RT				√	
Schouten Vn Der, 2007	Netherland	nRCT	-	237	BCS	237	Control	153	RT	√				
Silverstein, 2000	USA	nRCT	VNPI, margin status	209	BCS	209	RT	252	Control	√	√			√
Smith, 2006	Multicenter	nRCT	risk of cancer, age	1676	BCS	1676	RT	1733	Control	√			√	
Tunon-de-Lara, 2010	France	nRCT	-	67	Lumpectomy	67	Control	66	RT	√	√			
Wapnir, 2011	Multicenter	RCT	age	900	Lumpectomy	900	RT	899	RT+Tamoxifen	√	√			√
				403	Lumpectomy	403	Control	410	RT	√				√
Wärnberg, 2014	Sweden	RCT	-	526	BCS	526	RT	520	Control		√	√	√	√
Warren, 2005	Multicenter	nRCT	tumor size	626	BCS	626	RT	477	Control				√	√
Weng, 2000	USA	nRCT	-	24	LS	24	Control	38	RT	√				

Abbreviations: VNPI, Van Nuys Prognostic Index; BSC, breast-conserving surgery; LE, local excision; WLE, wide local excision; BSR, breast sector resection; LS, localized surgery; LC, local recurrence; M, metastasis; D, death; IBC, ipsilateral-breast cancer; CBC, contralateral breast cancer; RT, radiation therapy; RCT, randomized controlled trials; nRCT, non-randomized controlled trials

### Analysis based on both observational studies and RCTs

**Figure 3:**
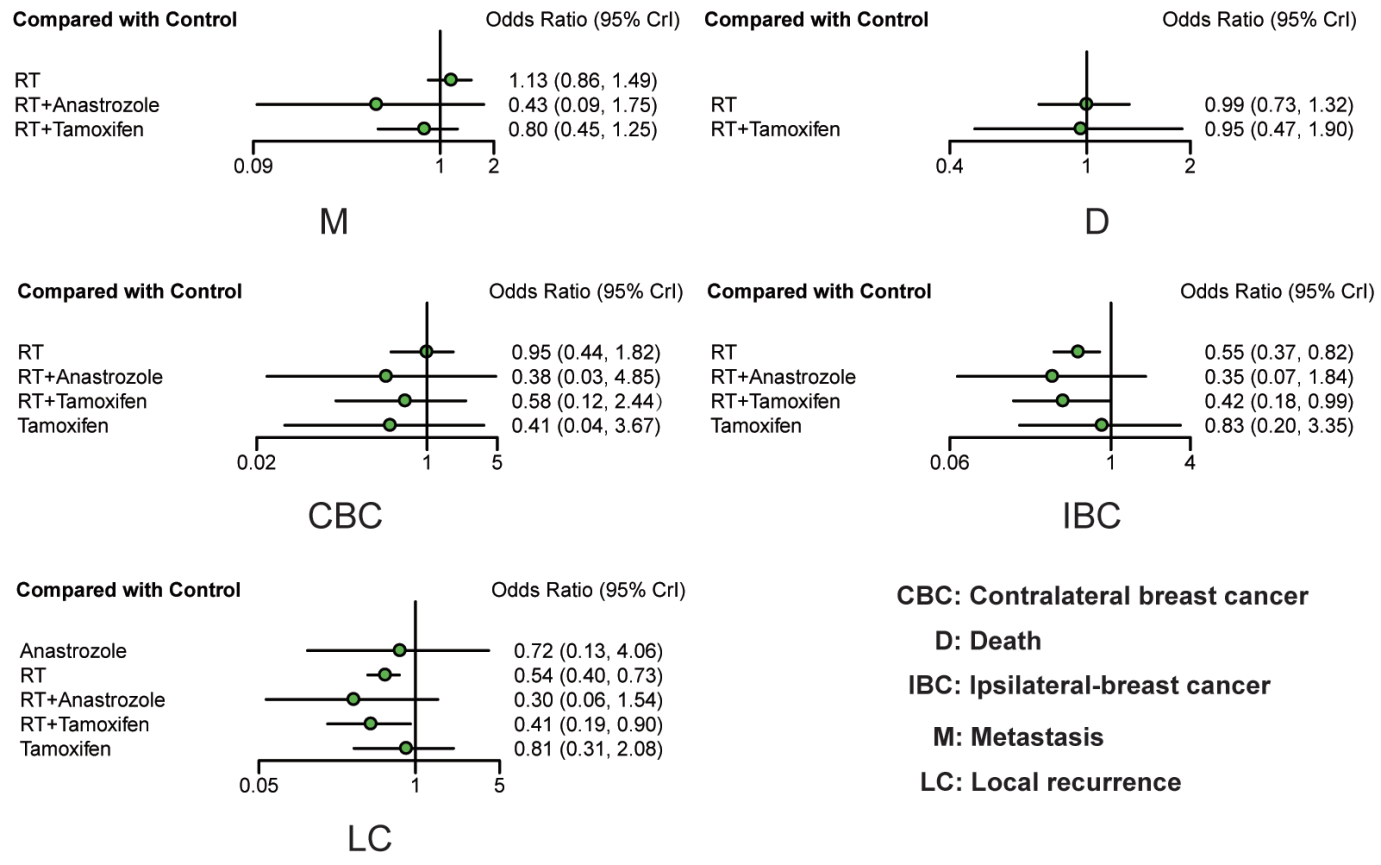
Forest plots of all outcomes based on observational studies and RCTs ORs with corresponding 95% CrIs were calculated to measure the relative efficacy of different treatments.

All eligible studies, including observational studies and RCTs, were included in the analysis procedure. The results are shown in the lower diagonal of Table 2 and in Figure 3. Patients treated by RT, and RT + tamoxifen were associated with a significantly decreased risk of LC compared to the control group (RT: OR = 0.54, 95% CrI = 0.40-0.73; RT + Tamoxifen: OR = 0.41, 95% CrI = 0.19-0.90). Similarly, RT and RT + tamoxifen were associated with a lower risk of IBC compared to the control group (OR = 0.55, 95%CrI = 0.37-0.82; OR = 0.42, 95%CrI = 0.18-0.99, respectively). No statistically significance differences were observed with regard to the outcomes CBC, D and M.

**Table 2:** Results of network comparison

LC							
**nRCT+RCT**	**Control**	0.99(0.19,6.23)	0.53(0.32,0.92)	0.22(0.04,1.22)	0.31(0.12,0.79)	1.12(0.43,3.46)	**RCT**
	0.72(0.13,4.06)	**Anastrozole**	0.53(0.09,2.92)	0.23(0.02,1.92)	0.31(0.05,1.62)	1.13(0.28,4.62)	
	0.54(0.40,0.73)	0.76(0.14,4.26)	**RT**	0.42(0.08,2.14)	0.59(0.25,1.32)	2.10(0.78,6.42)	
	0.30(0.06,1.54)	0.41(0.05,3.78)	0.55(0.11,2.77)	**RT+Anastrozole**	1.39(0.35,5.58)	4.95(0.97,29.37)	
	0.41(0.19,0.90)	0.57(0.11,3.06)	0.76(0.36,1.62)	1.39(0.33,5.81)	**RT+Tamoxifen**	3.60(1.46,10.07)	
	0.81(0.31,2.08)	1.13(0.27,4.81)	1.49(0.57,3.78)	2.75(0.51,14.15)	1.97(0.84,4.44)	Tamoxifen	
**CBC**							
**nRCT+RCT**	**Control**	1.48(1.14,1.92)	0.48(0.22,1.06)	0.76(0.44,1.31)	0.72(0.25,2.14)	**RCT**	
	0.95(0.44,1.82)	**RT**	0.33(0.15,0.69)	0.52(0.32,0.83)	0.49(0.17,1.43)		
	0.38(0.03,4.85)	0.39(0.03,4.95)	**RT+Anastrozole**	1.57(0.89,2.80)	1.48(0.50,4.57)		
	0.58(0.12,2.44)	0.61(0.17,2.27)	1.55(0.18,13.60)	**RT+Tamoxifen**	0.94(0.38,2.46)		
	0.41(0.04,3.67)	0.43(0.05,3.67)	1.12(0.07,16.78)	0.71(0.12,3.82)	**Tamoxifen**		
**IBC**							
**nRCT+RCT**	**Control**	0.47(0.27,0.84)	0.30(0.05,2.03)	0.36(0.13,1.01)	0.84(0.17,4.10)	**RCT**	
	0.55(0.37,0.82)	**RT**	0.64(0.11,3.97)	0.77(0.34,1.77)	1.77(0.33,9.49)		
	0.35(0.07,1.84)	0.64(0.13,3.16)	**RT+Anastrozole**	1.20(0.24,5.93)	2.77(0.23,32.79)		
	0.42(0.18,0.99)	0.77(0.36,1.63)	1.20(0.29,4.95)	**RT+Tamoxifen**	2.32(0.35,15.03)		
	0.83(0.20,3.35)	1.52(0.35,6.42)	2.36(0.27,20.09)	1.97(0.38,9.87)	**Tamoxifen**		
**M**							
**nRCT+RCT**	**Control**	1.14(0.84,1.57)	0.45(0.10,1.80)	0.84(0.44,1.38)	**RCT**		
	1.13(0.86,1.49)	**RT**	0.40(0.09,1.52)	0.73(0.42,1.07)			
	0.43(0.09,1.75)	0.38(0.09,1.48)	**RT+Anastrozole**	1.82(0.50,7.24)			
	0.80(0.45,1.25)	0.71(0.42,1.01)	1.84(0.50,7.39)	**RT+Tamoxifen**			
**D**							
**nRCT+RCT**	**Control**	1.02(0.79,1.32)	0.98(0.57,1.68)	**RCT**			
	0.99(0.73,1.32)	**RT**	0.97(0.59,1.55)				
	0.95(0.47,1.90)	0.96(0.52,1.79)	**RT+Tamoxifen**				

*Abbreviation: LC, local recurrence; M, metastasis; D, death; IBC, ipsilateral-breast cancer; CBC, contralateral breast cancer

### Analysis based on RCTs

**Figure 4:**
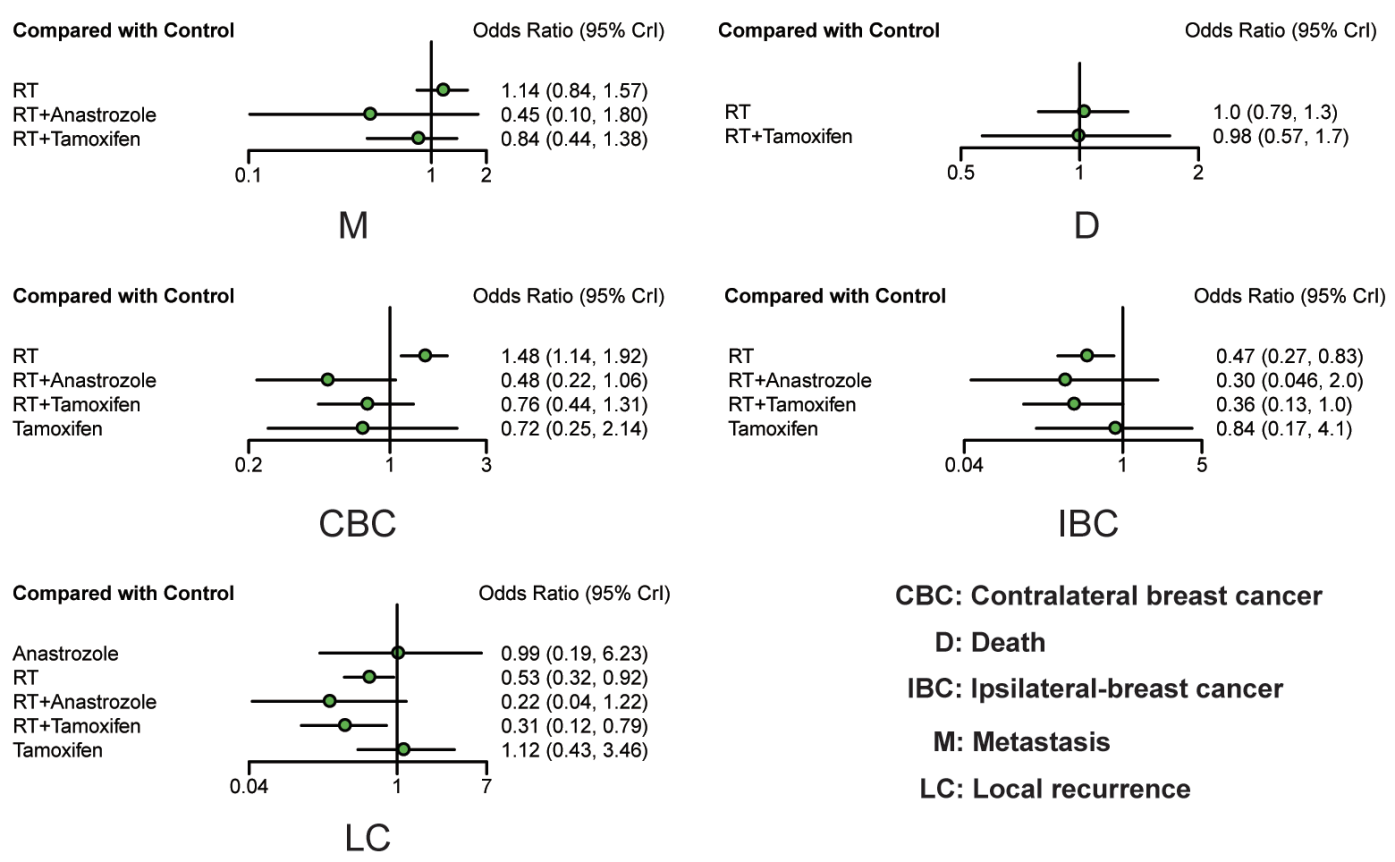
Forest plots of all outcomes based on RCTs ORs with corresponding 95% CrIs were calculated to measure the relative efficacy of different treatments.

In order to ensure that included observational studies did not introduce bias into this NMA, RCTs were selected from eligible studies and then analyzed using the same method. The corresponding results of our NMA are displayed in the upper diagonal of Table 2 and Figure 4. The results of the RCTs were similar to those of the observational studies, which indicated that the inclusion of observational studies introduced little heterogeneity to this NMA. Patients treated with RT and RT + tamoxifen exhibited a lower risk of LC compared to the control group (OR=0.53, 95% CrI = 0.32-0.92; OR = 0.31, 95% CrI = 0.12-0.79, respectively). Moreover, patients with RT+ tamoxifen also exhibited a lower risk of LC compared with tamoxifen alone (OR = 0.28, 95% CrI = 0.10-0.68). Additionally, RT was associated with a lower risk of IBC compared to the control group (OR = 0.47, 95% CrI = 0.27-0.84) and there was no statistically significant difference among different treatments in terms of M and D. However, RT was associated with a higher risk of CBC compared with the control group (OR = 1.48, 95% CrI = 1.14-1.92), RT+anastrozole (OR = 3.03, 95% CrI = 1.45-6.67) and RT+tamoxifen (OR = 0.92, 95% CrI = 1.20-3.13).

### Ranking results

According to the SUCRA presented in Table 3, the results between comparisons based on observational studies and RCTs and those based on RCTs alone were similar. RT + anastrozole ranked first in IBC, LC, M and CBC, and RT + tamoxifen also exhibited good performance in terms of IBC, LC, and M. In addition, tamoxifen was associated with a lower risk of CBC compared to all treatments except for RT + anastrozole, and besides RT + anastrozole and RT + tamoxifen, RT alone also effectively reduced the events of LC and IBC. However, no treatments exhibited outstanding performance with respect to D.

**Table 3:** Surface under the cumulative ranking curve (SUCRA)

nRCT+RCT					
	**CBC**	**D**	**IBC**	**LC**	**M**
**Anastrozole**	-	-	-	0.408	-
**Control**	0.26	0.45	0.128	0.152	0.359
**RT**	0.29	0.488	0.559	0.578	0.103
**RT_Anastrozole**	0.705	-	0.758	0.816	0.874
**RT_Tamoxifen**	0.561	0.561	0.736	0.754	0.664
**Tamoxifen**	0.684	-	0.318	0.292	-
**RCT**					
	**CBC**	**D**	**IBC**	**LC**	**M**
**Anastrozole**	-	-	-	0.285	-
**Control**	0.362	0.517	0.132	0.23	0.389
**RT**	0.027	0.44	0.578	0.587	0.111
**RT_Anastrozole**	0.92	-	0.755	0.89	0.866
**RT_Tamoxifen**	0.584	0.543	0.739	0.823	0.634
**Tamoxifen**	0.607	-	0.296	0.185	-

## DISCUSSION

The number of diagnosed cases of DCIS has increased due to the advent of new mammography techniques, and this increase has resulted in the challenge of tailoring post-surgery adjuvant therapies to individual patients. Some DCIS patients with low-risk lesions have been over-treated, while others with high-risk lesions have been under-treated. This may be associated with the recurrence or development of invasive breast cancer (IBC) [64]. Accordingly, differentiating post-surgery adjuvant therapies based on their efficacy may provide clinicians with assistance in order to overcome these issues.

This NMA evaluated five post-surgery treatments based on observational studies and RCTs. According to our results, treatments containing RT were effective in reducing the rate of LC and IBC. Moreover, RT + anastrozole and RT + tamoxifen were also associated with lower risk of M and CBC, which was confirmed by previous studies. As suggested by a long-term follow-up study, which focused on four randomized trials comparing lumpectomy with or without RT, radiation significantly reduced the local failure rate in DCIS patients [65]. Besides, two phase III trials indicated that tamoxifen with a dose of 20 mg/day reduced the risk of ipsilateral and contralateral events by approximately 30% [57, 58].

However, due to the lack of evidence, this NMA did not cover an analysis of safety, and it was reported that about 11%-31% patients treated with RT + tamoxifen did not complete the five-year tamoxifen treatment [66]. This poor rate of compliance may have been the result of long-term toxic effects associated with endometrial cancer or vascular thrombotic events. It was also reported that both RT and tamoxifen may trigger severe adverse events such as endometrial cancers, venous thromboembolism and secondary malignancies [67-69]. Therefore, long-term follow-up studies that consider the efficacy of RT or tamoxifen should be carried out in order to provide a more comprehensive suggestion for clinical practice.

Moreover, according to this NMA, RT + anastrozole seemed to be slightly more effective than RT + tamoxifen. Actually, aromatase inhibitors are potentially more effective than tamoxifen for preventing recurrence in postmenopausal females with invasive breast cancer and positive ER [57]. Various aromatase inhibitors have offered effective alternatives to tamoxifen, but the relative effectiveness of aromatase inhibitors compared to tamoxifen remains controversial. For instance, a randomized double-blinded trial (NSABP B-35) suggested that anastrozole significantly improved the breast cancer-free interval in DCIS patients under the age of 60 compared to the tamoxifen group, while another study reported no significant difference in the risk of overall recurrence between the anastrozole and tamoxifen group [70]. Therefore, although RT + anastrozole seemed to be more highly recommended than RT + tamoxifen, clinical decisions should be made based on several factors, including the patients’ physical characteristics, age, excision method and tolerance level.

Our study provides guidance for discriminating between the available post-surgery adjuvant therapies, however our NMA does contain some inherent limitations. First of all, although a large number of studies were included, their differences in follow-up times may increase the heterogeneity of this study. For example, adverse events such as LC, metastasis, progression to invasive breast cancers or death are rare events; therefore, the likelihood of the occurrence of such events in each study may be proportional to the duration. Studies with longer follow-up durations may contain more of these events. Having acknowledged this, our study did not adjust for duration when adverse events were taken into account. Apart from that, other variables, including age, margin and hormone-receptor status, may also increase the heterogeneity of patients and thus weaken the reliability of the results. Therefore, more detailed analyses should be conducted before certain conclusions are drawn. Most of the included studies cover the comparison of RT and placebo (control group). However, few trials include the analysis of anastrozole. Also, due to a lack of evidence, outcome information under certain treatments, including anastrozole and tamoxifen, was missing, and more comprehensive studies should be carried out in the future.

Overall, our study is an excellent start, as it demonstrates that patients treated with post-surgery adjuvant therapies had significant reduction in the risk of LC and IBC. Of all the treatments, RT + tamoxifen and RT + anastrozole were the two most recommended therapies. However, due to the lack of evidence demonstrating their safety, they should be applied with extra care.

## MATERIALS AND METHODS

### Systematic review and search for relevant studies

This study is based on a systematic and thorough review, which was conducted at an early stage in order to ensure the quality of our network meta-analysis. The review team included an experienced statistician, a clinician with expert knowledge and reviewers with competent search and information retrieval skills. Our review team also examined the corresponding research question in order to ensure that our study could produce meaningful results. The following aspects were considered in the systematic literature review: study population, comparators of our interest, clinical outcome definitions, study inclusion criteria, data extraction procedures, study quality assessment and the selection of an appropriate data analysis approach.

We performed an extensive literature search based on our systematic review, using the following medical subject headings (MeSH), in conjunction with their corresponding synonyms, to formulate a search strategy: DCIS of the breast, mastectomy (M), local excision (LE), BCS, breast sector resection (BSR), wide local excision (WLE), lumpectomy (L), localized surgery (LS), post-surgery adjuvant endocrine therapy, post-surgery radiation therapy (RT), efficacy. We included both observational trials and RCTs in this NMA. We searched a variety of databases, including Embase, MEDLINE/PUBMED, Cochrane Library, and Science Citation Index (SCI), according to the criteria mentioned above. Two reviewers carried out the entire search process independently of one another, and any disagreements were subject to third-party discussion.

### Studies selection

Retrieved articles were subject to the following eligibility criteria: 1) patients were at least 18 years old; 2) The diagnosis of DCIS must have been obtained by mammographic screening or histological examination; 3) Patients must have been treated by surgical procedures including mastectomy, M, L, LE, BCS, BSR, WLE, LS; 4) treatments must have contained at least two of the following treatments: tamoxifen, anastrozole, RT + tamoxifen, RT + anastrozole and placebo (control group); 5) outcomes must have included one of the following items, including LC, M, CBC, IBC and D; 6) Sufficient data for conducting a network meta-analysis could be obtained from studies. Studies which fell into one of the following categories were excluded: 1) duplicated studies; 2) meta-analysis or NMA; 3) reviews, abstracts, and case reports.

### Data extraction

Raw data were extracted from eligible studies using a data extraction spreadsheet, including study characteristics, baseline demographics and raw/summary statistics of the corresponding outcomes. The baseline characteristics of all trials were collected, including first author, publication year, country, study design, subgroup, sample size, follow-up time, surgery type, treatment, group size and outcomes.

### Statistical analysis

Software R 3.3.2 was used to conduct this Bayesian NMA and the random-effects assumption was adopted throughout our analysis due to the heterogeneous nature of included studies with respect to study design, population selection and following-up duration [71]. We used odds ratios (ORs) with 95% credible intervals to measure the relative efficacy of each treatment under different outcomes. Furthermore, the SUCRA was computed to obtain the relative ranking of different treatments. Moreover, in order to guarantee the reliability of this NMA, RCTs were selected from all eligible studies and then analyzed following the steps above.

## Abbreviations

DCIS: ductal carcinoma *in situ*
OR: odds ratio
SUCRA: surface under the cumulative ranking curve
RT: Radiotherapy
LC: local recurrence
IBC: ipsilateral breast cancer
